# Importance of Leucocytosis in Gynecology

**Published:** 1903-10

**Authors:** 


### IMPORTANCE OF LEUCOCYTOSIS IN GYNECOLOGY.

WEISS reviews 23 illustrative cases taken from Chrobak's clinic in Vienna, and from these concludes that the determination of the number of leukocytes forms in gynecology a substantial aid in differential diagnosis, in that constant leukocytosis with a number above 16,000 shows the presence of a suppurative process. If the process is of some duration, the number generally diminishes although noticeably high when medical advice was first sought. In cases of long standing, leukocytosis fails under any circumstances, and the physician in these cases must direct his treatment according to general principles determined by the general subjective condition of the patient and the objective condition of the genital tract. A negative condition of leukocytes in an acute illness or illness of short duration entirely excludes any suppurative process.—*Weiner Klinische Wochenschrift.*—*American Medicine.*

